# Tumor necrosis factor alpha and epidermal growth factor act additively to inhibit matrix gene expression by chondrocyte

**DOI:** 10.1186/ar1464

**Published:** 2004-11-29

**Authors:** Aaron R Klooster, Suzanne M Bernier

**Affiliations:** 1 CIHR Group in Skeletal Development and Remodeling, Department of Anatomy and Cell Biology, The University of Western Ontario, London, Ontario, Canada

**Keywords:** chondrocyte, epidermal growth factor, extracellular matrix, signaling, tumor necrosis factor alpha

## Abstract

The failure of chondrocytes to replace the lost extracellular matrix contributes to the progression of degenerative disorders of cartilage. Inflammatory mediators present in the joint regulate the breakdown of the established matrix and the synthesis of new extracellular matrix molecules. In the present study, we investigated the effects of tumor necrosis factor alpha (TNF-α) and epidermal growth factor (EGF) on chondrocyte morphology and matrix gene expression. Chondrocytes were isolated from distal femoral condyles of neonatal rats. Cells in primary culture displayed a cobblestone appearance. EGF, but not TNF-α, increased the number of cells exhibiting an elongated morphology. TNF-α potentiated the effect of EGF on chondrocyte morphology. Individually, TNF-α and EGF diminished levels of aggrecan and type II collagen mRNA. In combination, the effects of TNF-α and EGF were additive, indicating the involvement of discrete signaling pathways. Cell viability was not compromised by TNF-α or by EGF, alone or in combination. EGF alone did not activate NF-κB or alter NF-κB activation by TNF-α. Pharmacologic studies indicated that the effects of TNF-α and EGF alone or in combination were independent of protein kinase C signaling, but were dependent on MEK1/2 activity. Finally, we analyzed the involvement of Sox-9 using a reporter construct of the 48 base pair minimal enhancer of type II collagen. TNF-α attenuated enhancer activity as expected; in contrast, EGF did not alter either the effect of TNF-α or basal activity. TNF-α and EGF, acting through distinct signaling pathways, thus have additive adverse effects on chondrocyte function. These findings provide critical insights into the control of chondrocytes through the integration of multiple extracellular signals.

## Introduction

The role of epidermal growth factor (EGF) in the development of articular cartilage and the pathogenesis of arthritis is poorly understood. During development, EGF produced by the apical ectodermal ridge promotes the outgrowth of the limb bud mesoderm; however, migration away from the apical ectodermal ridge and downregulation of EGF expression in the mesodermal cells is necessary for differentiation of this cell population into chondrocytes [1]. We previously found that EGF encourages expansion of early committed chondrocytes but prevents the expression of link protein and aggrecan [2], two extracellular matrix components that are necessary for proper cartilage organization [3]. Proteoglycan accumulation is inhibited following treatment of mature articular chondrocytes with EGF in a monolayer or an organ culture [4,5]. We recently demonstrated an increase in proton efflux from chondrocytes treated with EGF resulting in localized acidification of the microenvironment that may contribute to altering both responsiveness of chondrocytes to extracellular stimuli and the activity of matrix metalloproteinases [6]. EGF is detectable in the synovial fluid of rheumatoid arthritis patients and influences the growth of synovial cells [7]. However, the effects on cartilage of EGF, alone or in conjunction with other mediators associated with inflammation, are poorly characterized.

Among the inflammatory mediators associated with joint diseases, tumor necrosis factor alpha (TNF-α) is well established as a key mediator in the progression of cartilage degeneration. High levels of TNF-α are detected in the synovial lining of rheumatic joints and in chondrocytes of osteoarthritic joints [8]. TNF-α promotes further expression of cytokines and chemokines by synovial cells and chondrocytes, thereby sustaining a renewal of local inflammatory mediators (reviewed in [9,10]). The presence of TNF-α correlates with a general loss of cartilage matrix molecules, such as type II collagen and aggrecan, due to increased production of matrix metalloproteinases and a reduction in synthesis of matrix molecules [11]. We recently demonstrated that activation of the NF-κB and mitogen-activated protein kinase (MAPK)/extracellular signal-regulated kinase (ERK) signaling pathways contributes to the TNF-α-mediated reduction of transcription of the type II collagen and link protein genes, as well as to a reduction in the steady-state mRNA levels of these key extracellular matrix components [12]. In rheumatic joints, elevated levels of EGF in the synovial fluid contribute to hyperplasia of the synovial lining, where synovial cells display increased expression of the EGF receptor ErbB-2 (also known as c-neu or HER2) [7,13,14] and amplify IL-1-mediated release of prostaglandin E_2 _from synovial cells [15]. However, the combined effects of EGF and TNF-α have not been investigated previously.

The objective of the present study was to determine whether EGF potentiates the response of chondrocytes to TNF-α. We investigated changes in chondrocyte morphology and function. The expression of type II collagen that is responsible for the structural integrity of articular cartilage and aggrecan that imparts resilience to the tissue were used as measures of chondrocyte function. Co-administration of TNF-α and EGF in the present study resulted in a marked increase in the proportion of elongated cells and an additive decrease in matrix gene expression. These changes in morphology and gene expression were found to be controlled in part by the MAPK pathway. Furthermore, EGF exerts its effects on matrix gene expression through a pathway independent of Sox-9.

## Materials and methods

### Primary cell culture

Articular chondrocytes were isolated from the distal femoral condyles of 1-day-old Sprague–Dawley rats (Charles River, St Hyacinthe, QC, Canada) as previously described [12]. The Animal Use Subcommittee of the University of Western Ontario Council on Animal Care approved the use of rats for these studies. Cells were plated at a density of 4.25 × 10^4 ^cells/cm^2 ^on tissue culture-treated plates (Falcon; BD Biosciences, Mississauga, ON, Canada) and cultured in RPMI 1640 media supplemented with 5% fetal bovine serum, 100 U/ml penicillin, 100 U/ml streptomycin and 10 mM HEPES (Invitrogen Life Technologies Inc., Burlington, ON, Canada). Culture media was replaced every 3 days. Culture medium was replaced with serum-free medium 16–20 hours prior to experiments.

Primary chondrocyte cultures were treated with TNF-α (30 ng/ml; Sigma Aldrich, Oakville, ON, Canada), with EGF (10 ng/ml; Sigma Aldrich) or with vehicle (phosphate-buffered saline + 0.01% bovine albumin; Roche Diagnostics, Laval, QC, Canada) in serum-free medium. These concentrations were previously found to elicit maximal responses from these cells [6,12]. For analysis of signaling pathways, cells were treated prior to addition of TNF-α or EGF with pharmacologic inhibitors including 2-[1-(3-dimethylaminopropyl)-1*H*-indol-3-yl]-3-(1*H*-indol-3-yl)-maleimide (10 μM bisindolylmaleimide [BIS] I, protein kinase C [PKC] inhibitor), or 2,3-*bis*(1*H*-indol-3-yl)-*N*-methylmaleimide (10 μM BIS V, inactive analog of BIS I), 1,4-diamino-2,3-dicyano-1,4-*bis*(2-aminophenylthio)-butadiene (10 μM U0126, mitogen-activated protein kinase kinase 1 and 2 [MEK1/2] inhibitor; Promega, Madison, WI, USA), and 1,4-diamino-2,3-dicyano-1,4-*bis*(methylthio)-butadiene (10 μM U0124, inactive analog of U0126). BIS I was used at a concentration that was greater than 500 times the inhibitory concentration 50% for conventional PKCs and twice the inhibitory concentration 50% for PKCζ. U0126 was used at a concentration previously found to be effective for inhibiting the phosphorylation of ERK1/2 [12]. The pharmacologic agents were obtained from EMD Biosciences (Calbiochem, La Jolla, CA, USA) unless otherwise stated.

### Imaging

Digital images of confluent monolayers were obtained using a Sony Power HAD 3CCD mounted onto a Nikon TMS inverted phase-contrast microscope (20 × objective magnification) (Nikon Canada Inc., Mississauga, ON, Canada). Images were acquired with NorthernEclipse V.5 software (Empix, Mississauga, ON, Canada). For the present study, an elongated cell was defined as having a predominant axis length exceeding three times the maximum width of the cell. The number of elongated cells per field of view (1.376 mm^2^) was counted and averaged.

### RNA extraction and northern blot analysis

Total RNA was collected from cells using the acid–guanidium–phenol–chloroform extraction method (Trizol; Invitrogen Life Technologies Inc.), according to the manufacturer's instructions. RNA was quantified by ultraviolet spectrophotometry. Total RNA (10 μg) was resolved on a 1.1% agarose gel containing formaldehyde. Equivalent loading of samples was verified by ethidium bromide staining before RNA was transferred to Nytran membranes (Schleicher & Schuell, Keene, NH, USA). RNA was fixed to the Nytran membrane by incubation at 80°C for 2.5 hours under vacuum. cDNA probes corresponding to the mouse C-propeptide of type II collagen (pKN225) [16], to 18S rRNA (DECAtemplate 18S mouse; Ambion, Austin, TX, USA), and to the C-terminus of rat aggrecan [17,18] were labeled with [α^32^P]dCTP (3000 Ci/mmol; Perkin Elmer, Woodbridge, ON, Canada) by a random-primed oligonucleotide method (Prime-a-gene labeling kit; Promega). Membranes were hybridized with cDNA probes and processed as described previously [19].

### Preparation of cell extracts and immunoblotting

Cell extracts were prepared as described previously [12]. Equivalent amounts of protein (15–30 μg) were resolved by electrophoresis on 7.5% polyacrylamide-SDS gels. Protein was transferred to nitrocellulose membrane (Schleicher & Schuell) by electroblotting. Transfer and equivalent loading was verified by subsequent staining with Ponceau Red (3-hydroxy-4-(2-sulfo-4-[4-sulfophenylazo]-phenylazo)-2,7-napthalenedisulfonic acid) [20]. Immunoblotting was performed by blocking the membrane for 1 hour with 5% non-fat milk (Carnation, North York, ON, Canada)/TBS 0.5% Tween. Membranes were incubated with antibodies for poly(ADP ribose) polymerase (PARP) (Santa Cruz Biotechnology, Santa Cruz, CA, USA), phospho-specific ERK1/2 (Anti-active MAPK; Promega) or ERK1 and ERK2 (Santa Cruz Biotechnology) according to the manufacturer's instructions. Target signals were detected with SuperSignal West Pico Chemiluminescent Substrate (Pierce Biotechnology Inc., Rockford, IL, USA) and exposure to Hyperfilm ECL (Amersham Biosciences, Baie d'Urfé, QC, Canada).

### Apoptosis analysis

Cells were seeded on Permanox chamber slides (Nalge Nunc, Naperville, IL, USA) at a density of 550 cells/mm^2^. Following treatment with factors, slides were fixed with 4% formalin solution. Apoptosis was assessed by the terminal deoxynucleotidyltransferase end-labeling with fluorescein-dUTP (TUNEL) method (Roche Diagnostics) as described in the manufacturer's instructions. Positive controls were treated for 10 min with DNAse I (Roche Diagnostics) to induce DNA breaks. Fluorescein activity was imaged by laser scanning confocal microscopy (LSM 510 Meta; Carl Zeiss Microscopy, Jena, Germany).

### MTT assay for cell viability

Cell viability was analyzed using the Cell Proliferation Kit I (MTT; Roche Diagnostics) following the manufacturer's instructions. Cells were seeded on 96-well plates at 400 cells/mm^2^, were cultured for 5 days and were then treated with TNF-α, or with EGF, or with TNF-α + EGF for an additional 24 hours. The colorimetric reaction was read on a μQuant spectrophotometer (Bio-Tek Instruments, Winooski, VT, USA) at 550 nm and 690 nm. The reading at 690 nm was used as a reference wavelength to calculate a corrected absorbance (A_550 _– A_690_).

### Transfections and luciferase reporter analysis

Chondrocytes were transfected with reporter constructs for NF-κB (Clontech, Palo Alto, CA, USA) or the type II collagen enhancer region (pGl3 4 × 48; a kind gift from Dr TM Underhill, The University of British Columbia, Vancouver, BC, Canada) [21]. Briefly, per transfection reaction, 0.1 μg reporter DNA and 2 ng PRL-SV40, a constitutively expressed renilla luciferase plasmid for monitoring transfection efficiency, were incubated with Fugene 6 transfection reagent (Roche Diagnostics). The mixture was added to a well of a 48-well plate and overlayed with 3.5 × 10^4 ^cells in serum-free culture medium. After 5 hours, medium containing serum was added to the wells. The following day, cells were treated with TNF-α (30 ng/ml), with EGF (10 ng/ml), with a combination of both or with vehicle in serum-free medium for 24 hours. The cells were lysed with 1 × Reporter Lysis Buffer (Promega) and luciferase activity quantified using the Dual Luciferase Assay System (Promega).

### Nuclear extract preparation and electrophoretic mobility shift assays

Isolation of nuclear extracts and the electrophoretic mobility shift assay were performed as previously described [12,22]. The double-stranded oligonucleotide containing the κB recognition sequence was purchased from Santa Cruz Biotechnology.

### Densitometry and statistical analysis

All data are representative of at least three independent experiments. Bands appearing on exposed film were analyzed using Kodak Digital Science software (Eastman Kodak, Rochester, NY, USA). Relative expression levels of type II collagen mRNA and aggrecan mRNA were standardized to the expression levels of 18S rRNA. One-way analysis of variance or repeated-measures analysis of variance followed by Tukey–Kramer post-test comparisons was performed to determine the statistical significance of differences among means (GraphPad Prism version 3.00; GraphPad Software, San Diego, CA, USA).

## Results

### Effects of TNF-α and EGF on chondrocyte morphology

**Figure 1:**
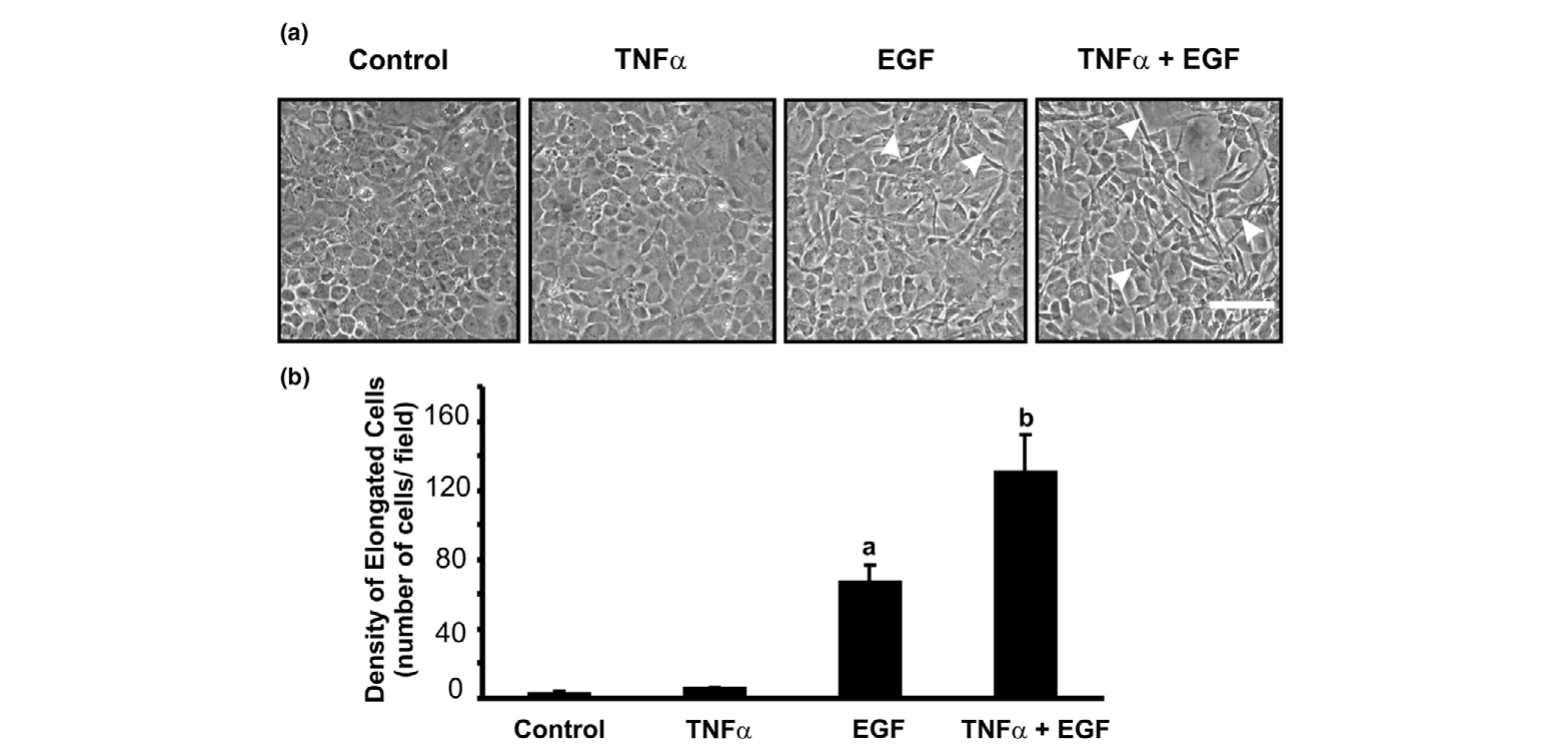
Tumor necrosis factor alpha (TNF-α) enhances elongated cell morphology induced by epidermal growth factor (EGF). Confluent monolayers of chondrocytes were treated with vehicle, TNF-α (30 ng/ml), EGF (10 ng/ml) or TNF-α + EGF for 24 hours. **(a) **Cell morphology was observed by phase contrast microscopy. Arrowheads indicate spinous processes that appear following incubation with EGF or TNF-α + EGF. An elongated cell is defined as having a predominant axis with a length exceeding three times the maximum width of the cell. Digital images of live cultures were captured at 20 × objective magnification. Bar = 100 μm. Images shown are representative of three independent experiments. **(b) **The total number of elongated cells per field (1.376 mm^2^) were counted, averaged for at least three independent experiments (*n *= 3–5), and analyzed by analysis of variance. ^a ^Significant difference from control (*P *< 0.01), ^b ^significant difference from control (*P *< 0.001) and significant difference from EGF-treated cells (*P *< 0.01).

The cellular morphology reflects the differentiation status of cells such as chondrocytes. For example, a change from a rounded to a more elongated morphology in response to EGF by CFK2 chondrocytic cells is associated with a diminished onset of expression of aggrecan and link protein gene [2]. To determine whether the morphology of primary chondrocytes expressing the matrix was affected by TNF-α or EGF, live cultures were examined by phase-contrast microscopy (Fig. 1a) and the number of elongated cells per field was quantified (Fig. 1b).

Previous studies established concentrations for TNF-α (30 ng/ml) [12] and EGF (10 ng/ml) [6] for maximal activation of signaling pathways in primary chondrocytes. Following a 24-hour treatment with vehicle (control) or TNF-α, the monolayers exhibited a 'cobblestone' appearance. In contrast, treatment with EGF promoted cell elongation, a change that was significantly potentiated by the presence of TNF-α. The distribution and arrangement of actin filaments were analyzed by phalloidin labeling. An increase in stress fibers was observed in elongated cells; however, the density of cells and prevalence of filamentous actin throughout the monolayer precluded any further quantitative analysis (data not shown).

### Effects of TNF-α and EGF on levels of aggrecan and type II collagen mRNA

**Figure 2:**
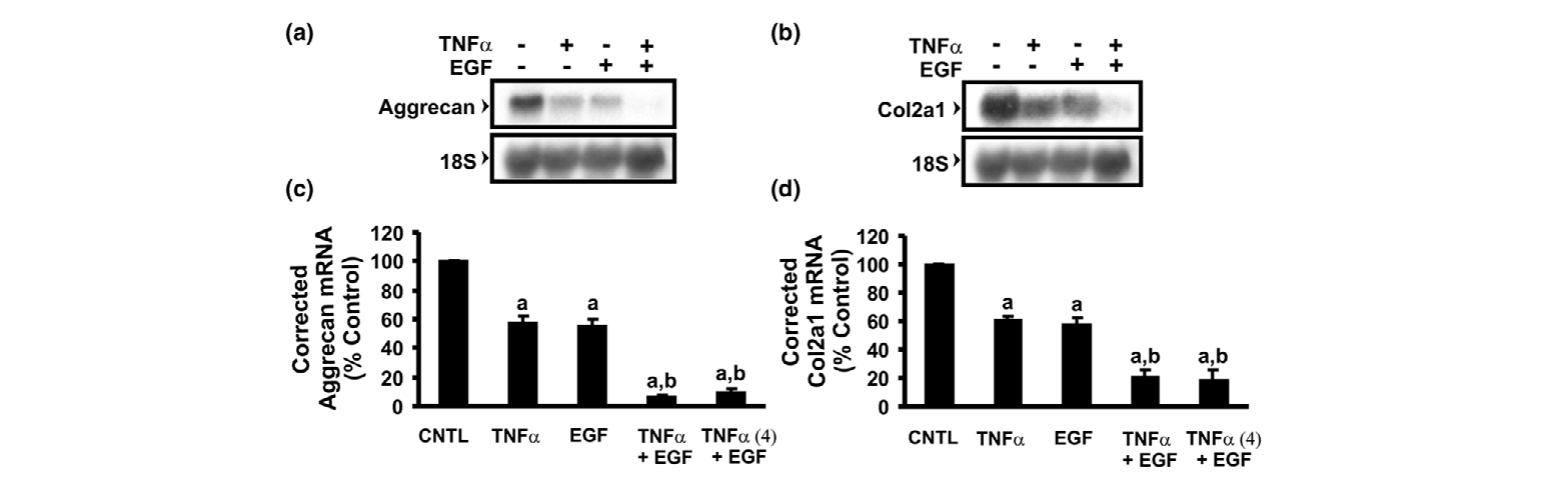
Tumor necrosis factor alpha (TNF-α) + epidermal growth factor (EGF) results in additive reduction in levels of aggrecan and type II collagen mRNA. Confluent monolayers of chondrocytes were treated for 24 hours with vehicle (CNTL), TNF-α (30 ng/ml), EGF (10 ng/ml) or TNF-α + EGF (*n *= 12). Levels of **(a) **aggrecan and **(b) **type II collagen mRNA were analyzed by northern blot of total RNA (10 μg). Changes in levels of **(c) **aggrecan and **(d) **type II collagen mRNA were quantified by densitometry. Levels were normalized to levels of 18S rRNA and data are expressed as the percentage of control ± standard error of the mean. ^a ^Significant difference from control (*P *< 0.001), ^b ^significant difference from TNF-α-treated and EGF-treated populations (*P *< 0.001).

We previously demonstrated that TNF-α reduces transcriptional expression of type II collagen and link protein genes [12]. In the present study, we characterized the effect of TNF-α on aggrecan mRNA levels and determined whether EGF altered type II collagen and aggrecan mRNA levels in the presence or absence of TNF-α. Cultures were treated with TNF-α or EGF individually or in combination (TNF-α + EGF) and the levels of aggrecan and type II collagen mRNA were analyzed (Fig. 2). Following 24 hours of treatment with TNF-α, levels of aggrecan and type II collagen mRNA were decreased by 42 ± 4% and 39 ± 2%, respectively. EGF alone decreased levels of aggrecan and type II collagen mRNA by 44 ± 5% and 42 ± 4%, respectively. Treatment of chondrocytes with TNF-α + EGF resulted in additive losses of aggrecan and type II collagen mRNA (93 ± 2% and 79 ± 4%, respectively). Treatment with TNF-α for 4 hours prior to the addition of EGF for the remainder of the 24 hours resulted in comparable decreases in levels of aggrecan and type II collagen mRNA (89 ± 2% and 81 ± 7%, respectively; data not shown). The combination of TNF-α and EGF therefore produces an additive decrease in both aggrecan and type II collagen mRNA levels, suggestive of discrete signals regulating mRNA expression by each factor.

### TNF-α and EGF do not alter the extent of apoptosis in the chondrocyte culture

**Figure 3:**
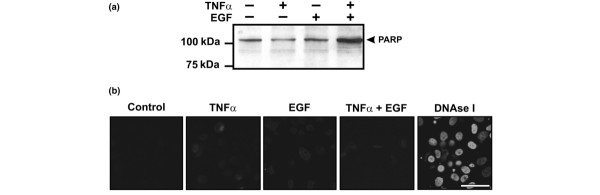
Apoptosis is not observed following tumor necrosis factor alpha (TNF-α) and/or epidermal growth factor (EGF) treatment. Confluent monolayers of chondrocytes were treated with vehicle, TNF-α (30 ng/ml), EGF (10 ng/ml) or TNF-α + EGF for 24 hours. **(a) **Early stages of apoptosis were assayed by immunoblot with an antibody specific for intact and cleaved forms of poly(ADP ribose) polymerase (PARP). No cleavage of PARP (i.e. appearance of a band at 89 kDa) was detected following any of the treatments. Blot shown is representative of three independent experiments. **(b) **Apoptosis-induced DNA strand breaks were examined by *in situ *labeling (terminal deoxynucleotidyl transferase-mediated dUTP nick end-labeling [TUNEL]) and imaged using confocal microscopy. No TUNEL labeling was detected with any of the treatments. Cells treated with DNAse I to induce DNA breaks served as a positive control. Bar = 50 μm. Images are representative of three independent experiments.

Cultures treated with TNF-α, with EGF or with TNF-α + EGF were assessed for evidence of apoptosis using an early marker, PARP (Fig. 3a). PARP is a 116 kDa protein involved in DNA repair [23] that is cleaved as part of the caspase cascade initiated in cells undergoing apoptosis. Cell extracts were immunoblotted for the presence of intact and cleaved forms of PARP. Neither loss of intact PARP nor the appearance of cleaved moieties (85 kDa) was detected following 24 hours of treatment with TNF-α, with EGF or with TNF-α + EGF. Interestingly, TNF-α + EGF increased the amount of PARP present in the chondrocytes. To confirm the lack of apoptosis in factor-treated cultures, the presence of DNA strand breaks was evaluated by *in situ *labeling (TUNEL) (Fig. 3b). TUNEL labeling was not detected following any of the treatments.

**Figure 4:**
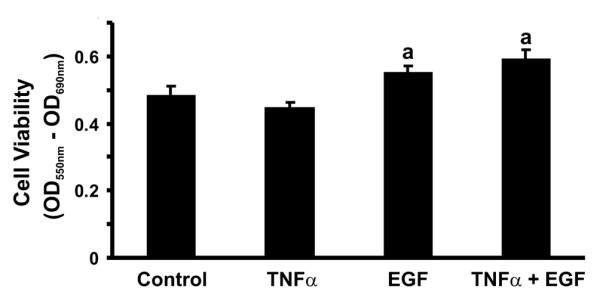
Cell viability is maintained in the presence of tumor necrosis factor alpha (TNF-α) and/or epidermal growth factor (EGF). Subconfluent monolayers of chondrocytes were treated with vehicle, TNF-α (30 ng/ml), EGF (10 ng/ml) or TNF-α + EGF for 24 hours. Cell viability was assessed by MTT assay. Data shown are combined from four independent experiments, presented as the mean corrected absorbance ± standard error of the mean analyzed by repeated-measures analysis of variance. ^a ^Significant difference from control (*P *< 0.01, *n *= 4). OD, optical density.

Cell viability was also assessed using the MTT assay (Fig. 4). TNF-α did not significantly alter cell viability after 24 hours. EGF caused an increase in metabolism of the tetrazolium salt at 24 hours that was not, however, changed significantly by co-addition of TNF-α, probably reflecting an increase in chondrocyte number. These results suggest that reduction in aggrecan and type II collagen mRNA levels induced by TNF-α and EGF are not correlated with initiation of programmed cell death (Fig. 3) or a decrease in cell number (Fig. 4).

### EGF does not alter NF-κB activation by TNF-α

**Figure 5:**
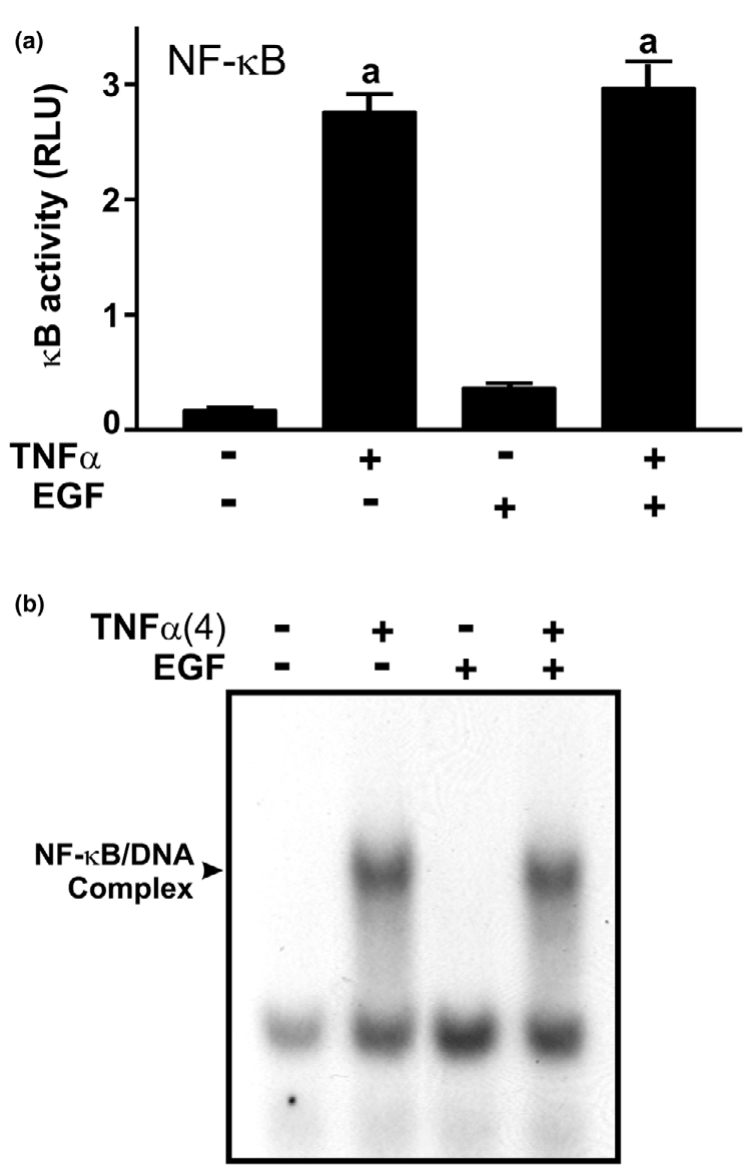
Epidermal growth factor (EGF) does not alter NF-κB activation by tumor necrosis factor alpha (TNF-α). **(a) **Chondrocytes transfected with a κB reporter were treated with TNF-α (30 ng/ml), EGF (10 ng/ml) or TNF-α + EGF. Reporter activity was assessed after 24 hours. Data were corrected for a constitutively expressed reporter (pSV40-RL), presented as mean relative luciferase units (RLU) ± standard error of the mean, and analyzed using one-way analysis of variance followed by a Tukey–Kramer post-test. ^a ^Significant difference from basal κB activity at *P *< 0.001 and no significant difference between TNF-α and TNF-α + EGF. **(b) **The effect of EGF on sustained NF-κB activity induced by TNF-α was determined by treating confluent chondrocytes with TNF-α (30 ng/ml) alone for 24 hours, EGF (10 ng/ml) alone for 20 hours, or TNF-α for 4 hours followed by the addition of EGF (10 ng/ml) for co-treatment for a further 20 hours. Nuclear extracts were prepared and analyzed by electrophoretic mobility shift assay for activation of NF-κB. The reporter assay and band shift shown are representative of three independent experiments.

Several signaling pathways known to mediate the effects of TNF-α and EGF were next investigated. We previously demonstrated that primary articular chondrocytes treated with TNF-α exhibit sustained activation of NF-κB at 24 hours, and that NF-κB partially mediated the reduction in type II collagen mRNA induced by TNF-α [12]. To assess whether changes in NF-κB activity contribute to the observed decrease in aggrecan and type II collagen mRNA, chondrocytes were transfected with a κB-driven reporter to detect functional activation of NF-κB (Fig. 5a). As expected, TNF-α significantly increased reporter levels. In contrast, EGF did not activate NF-κB or alter activation of NF-κB by TNF-α. Furthermore, sustained NF-κB activation induced by TNF-α was unchanged by EGF as determined by the electrophoretic mobility shift assay (Fig. 5b). The heightened decrease in aggrecan and type II collagen mRNA induced by TNF-α + EGF was therefore not the result of altered NF-κB activation.

### Inhibition of PKC does not prevent reduction in levels of aggrecan or type II collagen mRNA by TNF-α and EGF

**Figure 6:**
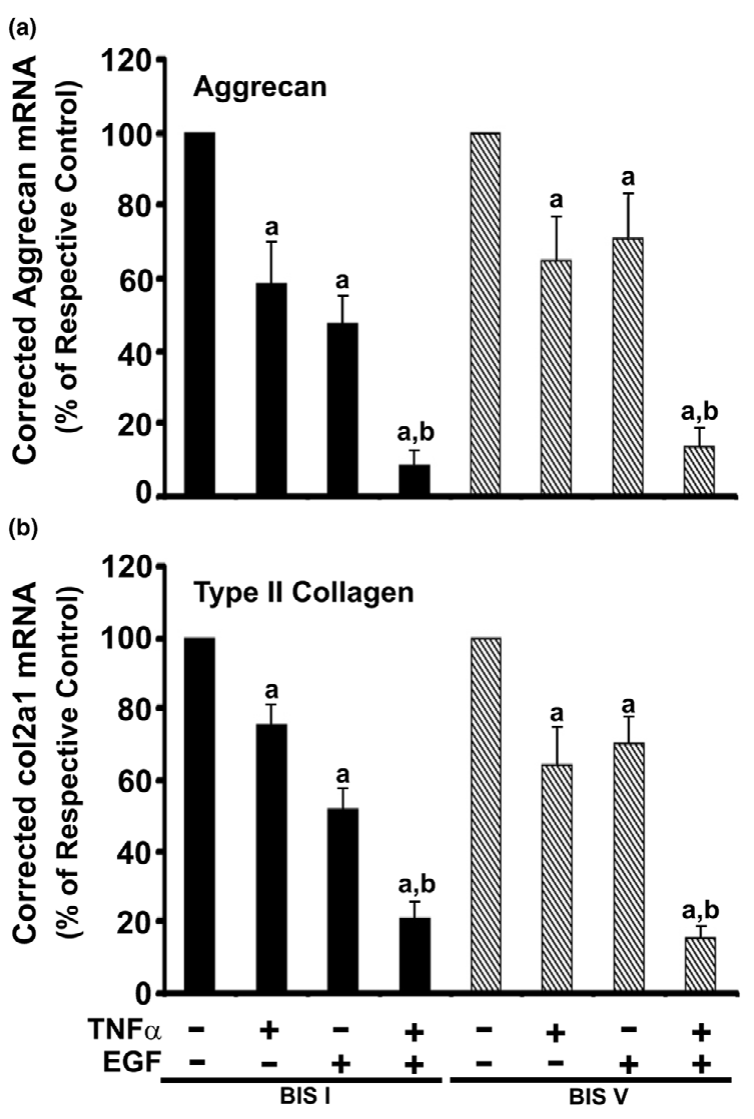
Inhibition of protein kinase C does not prevent reduction in levels of aggrecan or type II collagen mRNA by tumor necrosis factor alpha (TNF-α) and epidermal growth factor (EGF). Confluent monolayers of chondrocytes were pretreated with 10 μm bisindolylmaleimide (BIS) I or the structurally related, nonfunctional analog BIS V (10 μm) for 15 min, followed by treatment with TNF-α (30 ng/ml), EGF (10 ng/ml) or TNF-α + EGF in combination for 24 hours. Levels of **(a) **aggrecan and **(b) **type II collagen mRNA were determined by northern blot analysis of total RNA (10 μg). Levels were normalized to levels of 18S rRNA and data are expressed as the percentage of respective controls ± standard error of the mean (*n *= 4–6). ^a ^Significant difference from respective control populations (*P *< 0.05), ^b ^significant difference from populations treated individually with TNF-α or EGF (*P *< 0.001).

TNF-α and EGF have been found to activate PKC in other cell types. The role of PKC signaling in the reduction of aggrecan and type II collagen mRNA by TNF-α and EGF was examined using a pharmacologic inhibitor of PKC (Fig. 6). Cultures were pretreated with the PKC inhibitor BIS I at a concentration known to inhibit activation of several PKC isoforms, specifically PKCα, PKCβ_I_, PKCβ_II_, PKCγ, PKCδ, PKCε, and PKCζ [24,25], or with BIS V, an inactive analog of BIS I. TNF-α and/or EGF were added and the mRNA levels were analyzed by northern blot. Pretreatment with either BIS I or BIS V did not prevent the reduction in levels of aggrecan and type II collagen mRNA by TNF-α, by EGF or by TNF-α + EGF. Activation of PKC thus does not appear to be involved in the regulation of matrix gene expression by TNF-α and EGF. Neither BIS I nor BIS V treatment alone significantly altered the levels of aggrecan and type II collagen mRNA.

### Changes in cell morphology induced by EGF or the combination of TNF-α and EGF are suppressed by inhibition of MAPK

**Figure 7:**
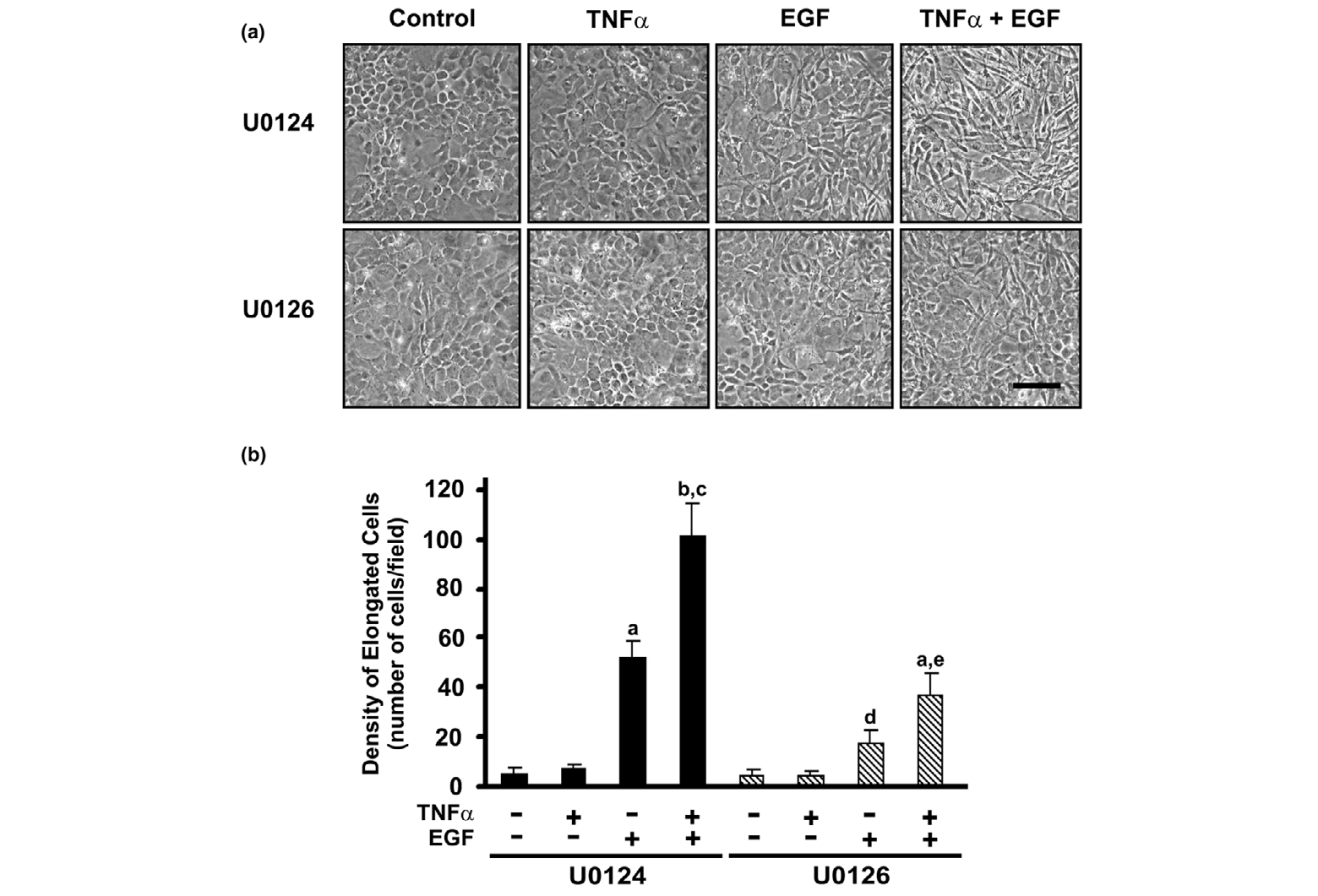
Changes in cell morphology induced by the combination of tumor necrosis factor alpha (TNF-α) + epidermal growth factor (EGF) are partially reduced by inhibition of MEK1/2. **(a) **Confluent monolayers of chondrocytes were pretreated with U0124 (10 μM) or U0126 (10 μM) for 15 min followed by 24-hour treatment with TNF-α (30 ng/ml), EGF (10 ng/ml) or TNF-α + EGF. Digital images of live cultures were captured at 20 × objective magnification. Bar = 100 μm. Images are representative of two independent experiments. **(b) **An elongated cell is defined as having a predominant axis with a length exceeding three times the maximum width of the cell. The total number of elongated cells per field (1.376 mm^2^) were counted, averaged for three independent experiments (*n *= 3), and analyzed by analysis of variance. ^a ^Significant difference from respective control (*P *< 0.05), ^b ^significant difference from respective control (*P *< 0.001), ^c ^significant difference from U0124 + EGF-treated cells (*P *< 0.01), ^d ^significant difference from U0124 + EGF-treated cells (*P *< 0.05), ^e ^significant difference from U0124 + TNF-α + EGF-treated cells (*P *< 0.001).

EGF is a well-characterized activator of the MAPK/ERK pathway [26]. We investigated whether the changes observed in cell morphology were dependent on the MAPK/ERK pathway. Chondrocytes were treated with the selective inhibitor of MEK1/2 activation, U0126, at a concentration previously found to inhibit ERK1/2 phosphorylation in these cells [12], or the inactive analog U0124 followed by TNF-α and/or EGF or vehicle. After 24 hours, the chondrocytes treated with U0124 or with U0126 followed by treatment with either vehicle or TNF-α exhibited similar morphology (Fig. 7a) to that observed in the absence of pharmacological agents (Fig. 1).

The number of elongated cells per field was also counted (Fig. 7b). The number of elongated cells induced by EGF and by TNF-α + EGF was markedly reduced following pretreatment with U0126. Cultures treated with U0124 followed by EGF or by TNF-α + EGF exhibited changes in the number of elongated cells comparable with vehicle-pretreated cultures. Changes in morphology in response to EGF and to TNF-α + EGF are thus dependent on a MEK1/2-regulated process.

### Inhibition of the MAPK pathway prevents TNF-α and EGF-mediated loss of aggrecan and type II collagen mRNA

**Figure 8:**
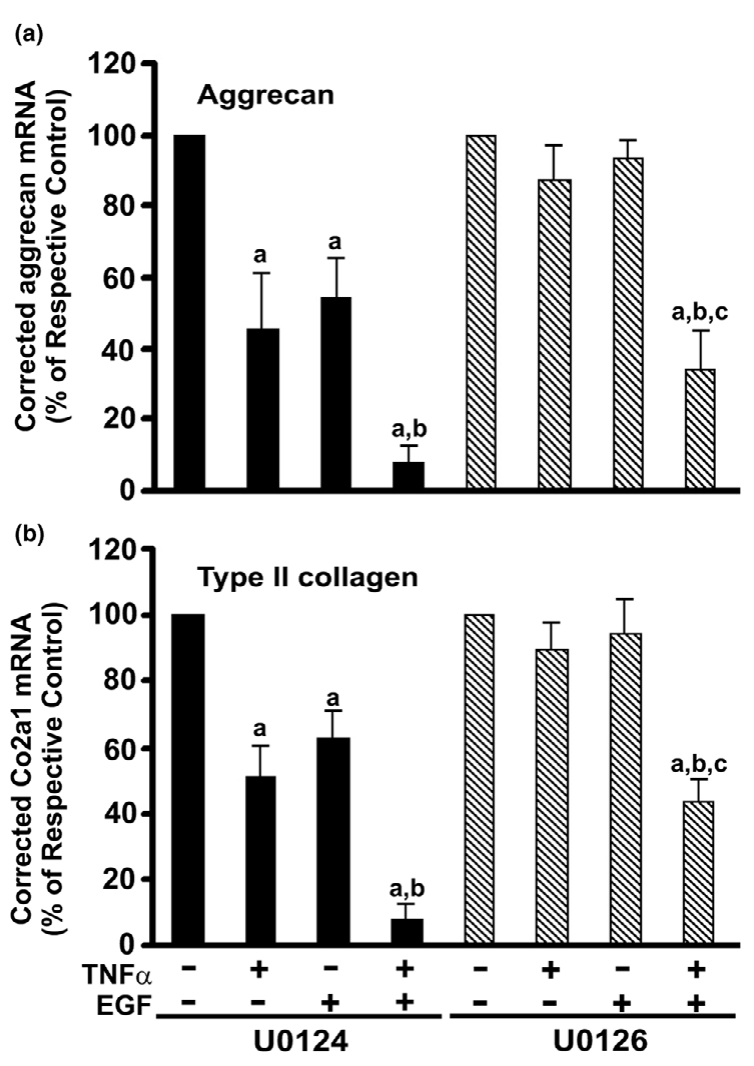
Inhibition of the mitogen-activated protein kinase pathway prevents tumor necrosis factor alpha (TNF-α) and epidermal growth factor (EGF)-mediated loss of aggrecan and type II collagen mRNA. Confluent chondrocytes were pretreated with U0124 (10 μm, inactive analog of U0126) or U0126 (10 μm, a MEK1/2 inhibitor), for 15 min, followed by treatment with TNF-α (30 ng/ml), EGF (10 ng/ml) or TNF-α + EGF for 24 hours. Levels of **(a) **aggrecan and **(b) **type II collagen mRNA were assessed by northern blot analysis of total RNA (10 μg). Levels were normalized to levels of 18S rRNA and data are expressed as the percentage of respective control ± standard error of the mean (*n *= 5). ^a ^Significant difference from respective control (*P *< 0.001), ^b ^significant difference from cultures treated individually with TNF-α or EGF (*P *< 0.01), ^c ^significant difference from cultures treated with U0124 followed by addition of TNF-α + EGF (*P *< 0.05).

We previously demonstrated that activation of the MAPK signaling cascade contributed to a reduction in type II collagen mRNA levels [12]. The involvement of the MAPK/ERK pathway in the reduction in aggrecan and type II collagen mRNA levels by EGF and TNF-α was investigated. Cells were pretreated with U0124 or U0126 followed by the addition of TNF-α and/or EGF or vehicle for 24 hours (Fig. 8). Cultures treated with the inactive inhibitor exhibited no change in either the basal levels of mRNA (data not shown) or the extent of reduction in aggrecan or type II collagen mRNA levels from that of untreated cultures (Fig. 2). U0126 prevented the losses mediated by the individual factors and partially protected against the effect of TNF-α and EGF in combination.

**Figure 9:**
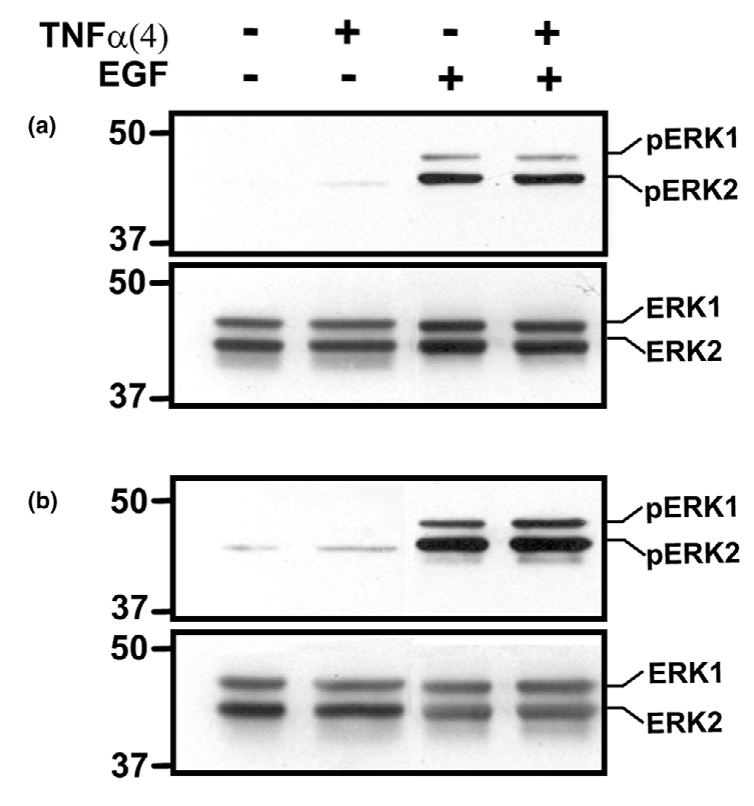
Comparable levels of extracellular signal-regulated kinase (ERK)1/2 phosphorylation are observed in chondrocytes treated with epidermal growth factor (EGF) alone or in combination with tumor necrosis factor alpha (TNF-α). Confluent monolayers of chondrocytes were treated for 4 hours with TNF-α (30 ng/ml) followed by **(a) **15 min treatment or **(b) **30 min treatment with EGF (10 ng/ml). Phosphorylation of ERK1/2 was determined by immunoblot assay using phospho-specific ERK1/2 antibody and ERK1 antibody (antibody against ERK1 is cross-reactive for ERK2). Blots shown are representative of three independent experiments.

To determine the MAPK responsiveness of chondrocytes to the combination of these factors, the phosphorylation of ERK1/2 was assessed. Cell extracts were collected from cultures treated for 4 hours with vehicle or with TNF-α prior to the addition of EGF, and were immunoblotted with antibody specific for phosphorylated forms of ERK1/2 (Fig. 9). In a previous study [12], we demonstrated phosphorylation of ERK1/2 within 15 min of the addition of TNF-α. In the present study, we found that phosphorylation of ERK1/2 returned to control levels after 4 hours of treatment with TNF-α. Phosphorylation of both ERK1 and ERK2 by EGF was apparent after 5 min and had not diminished by 30 min in the vehicle-treated cells. As both simultaneous and sequential addition of TNF-α and EGF produced comparable reductions in matrix gene mRNA levels, the MAPK response to EGF was assessed in the presence or absence of a 4-hour TNF-α pretreatment. Cultures that received TNF-α pretreatment followed by EGF had levels of ERK1/2 phosphorylation comparable with those cultures treated with EGF alone. An increase in the level of phosphorylation therefore did not contribute to the greater loss of matrix gene mRNA expression.

### Effects of EGF on type II collagen mRNA are independent of the Sox-9 response region of the type II collagen enhancer

**Figure 10:**
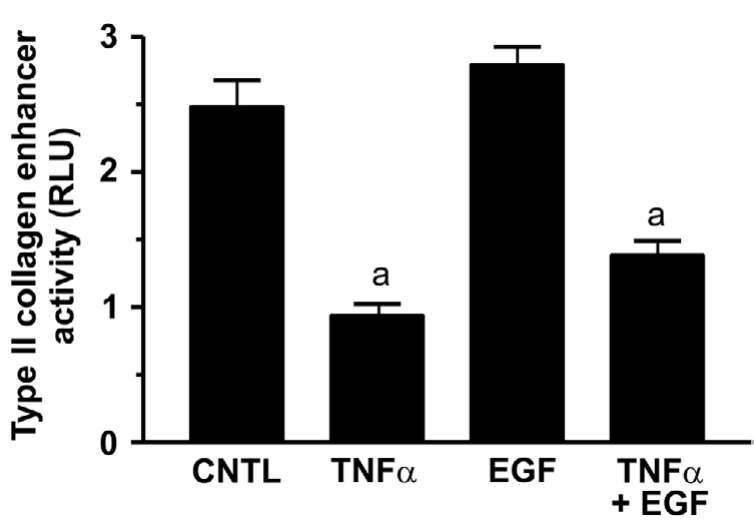
Epidermal growth factor (EGF) and tumor necrosis factor alpha (TNF-α) differentially regulate the activity of the type II collagen enhancer. Chondrocytes were co-transfected with the type II collagen enhancer luciferase reporter and the pSV40-RL constructs. The transfected cells were treated with vehicle (CNTL), TNF-α (30 ng/ml), EGF (10 ng/ml) or TNF-α + EGF, and the reporter activities were analyzed after 24 hours. Values of relative luciferase expression (corrected for transfection efficiency) were compared using one-way analysis of variance followed by a Tukey–Kramer post-test, and are presented as the mean ± standard error of the mean. ^a ^Significantly different from others at *P *< 0.001. Data are representative of three independent experiments. RLU, relative luciferase units.

Expression of both type II collagen and aggrecan genes is regulated by a transcriptional complex containing members of the Sox family, namely Sox-5, Sox-6, and Sox-9 [27,28]. A Sox-9 regulatory element resides in the type II collagen enhancer. To determine whether the reduction in type II collagen mRNA levels by EGF involves the minimal enhancer region, chondrocytes were transfected with a reporter construct for this 48 base pair region [21] and were treated with TNF-α and/or EGF or with vehicle (Fig. 10). As previously demonstrated [12], TNF-α markedly reduced activity at this regulatory region consistent with impairment of Sox-9 binding or activity. In contrast, EGF did not alter the activity of the enhancer region and the effect of TNF-α + EGF was not significantly different from that of TNF-α alone. These results indicate that the EGF-mediated reduction in levels of type II collagen mRNA is independent of the minimal enhancer regulatory region and, therefore, probably independent of Sox-9 regulation.

## Discussion

The morphology of cells is regulated by extracellular signals including soluble mediators and a matrix. Moreover, a relationship exists between the morphology and the state of differentiation. In the present study we investigated the effects of TNF-α, a factor that did not alter cell morphology, and the effects of EGF, a factor that induced a change in cell morphology. It is well established that removal of chondrocytes from their environment rich in extracellular matrix to a two-dimensional culture causes a change in morphology from rounded/cuboidal to more flattened and spread cells [29]. These morphological changes are accompanied by changes in the organization of the actin cytoskeleton [30]. Coincident with the change in chondrocyte shape is a loss of expression of phenotypic markers such as type II collagen and aggrecan [29,31,32], a phenomenon referred to as dedifferentiation. In addition, non-matrix factors can influence the organization of the actin cytoskeleton and can have profound effects on differentiation of chondrocytes. For example, bone morphogenetic protein-7 and IL-1 promote and restrict chondrogenesis, respectively, through changes in the distribution of focal adhesion proteins that are essential components of the cytoskeletal complexes. Their induction or their repression, respectively, of type II collagen gene expression involves altering the organization of the actin cytoskeleton [33]. In the present study, however, while only EGF induced a notable change in cell morphology, both TNF-α and EGF brought about comparable reductions in the mRNA levels of cartilage matrix genes. Morphological changes may thus be linked to expression of a differentiated phenotype for some inflammatory mediators.

Cell survival is essential for ensuring ongoing homeostatic maintenance of cartilage and for bringing about repair to damaged cartilage. Maintaining integrity of the nuclear material is critical, and the repair of damaged DNA is dependent on PARP. When a cell initiates apoptosis, PARP is targeted by caspase 3 and caspase 7, and is cleaved, rendering the enzyme inactive (properties of PARP are reviewed in [34,35]). Furthermore, in a caspase-independent manner, overactivation of PARP can lead to cell death through the release of apoptosis-inducing factor [35]. In the present study, apoptosis was not initiated by TNF-α and/or EGF as there was no cleavage of PARP and no evidence of DNA fragmentation (TUNEL staining). TNF-α and EGF separately had no effect on levels of PARP; however, when TNF-α and EGF were combined, increased levels of PARP were found. It is not clear whether the increase in PARP is due to increased *de novo *synthesis or to prevention of turnover. A similar phenomenon has been observed in retinal tissue following ischemia-reperfusion injury [36], another situation in which multiple inflammatory mediators are present. The upregulation of PARP by the combination of TNF-α and EGF suggests a protective response by chondrocytes as a certain cellular threshold for tolerance is exceeded. Furthermore, PARP can mediate transcriptional suppression through direct interaction with promoter DNA or modification of regulatory transcription factors such as NF-κB. Further investigation would be needed to determine whether PARP is involved in the reduced mRNA levels of type II collagen and aggrecan.

TNF-α and EGF activate several intracellular signaling pathways through their respective receptors or via cross-talk of pathway components. The concentrations of factors used in this study are sufficient to elicit maximal responses in these chondrocytes [6,12]. The additive nature of the decrease in aggrecan and type II collagen mRNA suggests the involvement of at least two signaling pathways activated by TNF-α and EGF. We have previously shown that TNF-α does not activate p38 in this system [12]. Furthermore, NF-κB activity has been implicated in mediating the effects of TNF-α and IL-1β on the expression of type II collagen [12,37]. Disruption of the actin cytoskeleton with cytochalasin D or with latrunculin B results in an increase in NF-κB activation [38]. Although inducing a change in morphology, EGF did not alter the activity of NF-B, either basal or that induced by TNF-α. Similarly, PKC is typically activated in response to TNF-α or EGF and can mediate an activation of MAPK signaling [39,40]. In the present study, however, inhibition of several isoforms of PKC did not alter the observed losses in aggrecan and type II collagen mRNA. The pharmacological inhibitor of MEK1/2 suppressed mRNA loss and changes in cell morphology. The MAPK/ERK pathway is thereby at least partially involved in regulating the aggrecan and type II collagen genes and in remodeling of the cytoskeleton in response to factors present during inflammation.

The MAPK/ERK pathway plays an important role in directing alterations of the cytoskeleton. For example, constitutively active MAPK induces morphological changes in fibroblasts, coinciding with disruption of stress fibers and disappearance of focal adhesions [41]. The MEK/ERK pathway is crucial in the control of hepatocyte cell morphology and cell cycle in response to EGF [42]. In chondrocytes, the MAPK/ERK pathway may have dual function in controlling the alteration in gene expression during cartilage degeneration and cytoskeletal remodeling. Induction of dedifferentiation may be a consequence of proliferation induced by growth factors, a process involving MAPK that may shift the balance away from differentiated phenotype towards amplification of the population. When both TNF-α and EGF are present, inhibition of MEK1/2 failed to completely prevent a reduction in mRNA levels of matrix components. The level of ERK1/2 phosphorylation induced by EGF was not altered in the presence of TNF-α, suggesting that MEK1/2 activity was also not altered and could be fully inhibited by the concentration of U0126 used. Taken together, these data suggest that although blockade of MEK1/2 can prevent the loss of aggrecan and type II collagen mRNA by TNF-α and EGF individually, additional signals beyond the MAPK pathway are probably involved when the factors are combined.

The intracellular signals that control matrix gene expression elicit their effects through regulation of gene transcription or through post-transcriptional modification and turnover of gene products (i.e. stability of mRNA). A key molecule involved in the transcriptional regulation of both type II collagen and aggrecan is Sox-9 [28,43,44]. Sox-9 acts by binding to enhancer regions of the type II collagen gene and to regulatory regions of the aggrecan gene to drive promoter activity [43]. Although the exact mechanism of loss of aggrecan mRNA in response to TNF-α and EGF remains unclear, the lack of change in activity at the type II collagen enhancer in response to EGF suggests that changes in Sox-9 activity do not mediate the EGF effects. Furthermore, these results together with the fact that the MAPK/ERK pathway was activated by EGF in this system suggest that this region is independent of MAPK/ERK activity. There are, however, alternate sites within these genes that may govern expression, such as SP1/SP3, C-Krox, and Stat1 [45-48]. In addition, activation of signaling pathways can increase synthesis of proteins responsible for the breakdown of existing mRNA, thereby increasing mRNA turnover. In this regard, we previously demonstrated that TNF-α reduces levels of type II collagen mRNA by approximately 40% when transcription was fully inhibited pharmacologically [12]. The additional loss of mRNA species following the treatment with both EGF and TNF-α in the present study suggests that intracellular signals target regulatory elements external to the enhancer-like sequence or stability of the mRNA.

## Conclusion

In this study, changes in chondrocyte phenotype and function were determined following treatment with TNF-α and EGF, mediators that contribute to sustaining the inflammatory processes associated with arthritis. The effects of this combination of factors have not been explored previously in cartilage or in other tissues. The expression of matrix genes critical for maintaining structural and functional integrity of cartilage was downregulated additively by TNF-α and EGF through mechanisms that involved at least two signals convergent on matrix gene regulation. Multiple inflammatory mediators can therefore profoundly reduce chondrocyte function and contribute to the progression of cartilage degeneration through several distinct signaling events.

## Abbreviations

BIS = bisindolylmaleimide; EGF = epidermal growth factor; ERK = extracellular signal-regulated kinase; IL = interleukin; MAPK = mitogen-activated protein kinase; MEK1/2 = mitogen-activated protein kinase kinase 1 and 2; NF = nuclear factor; PARP = poly(ADP ribose) polymerase; PKC = protein kinase C; TNF-α = tumor necrosis factor alpha; TUNEL = terminal deoxynucleotidyl transferase-mediated dUTP nick end-labeling; U0124 = 1,4-diamino-2,3-dicyano-1,4-*bis*(methylthio) butadiene; U0126 = 1,4-diamino-2,3-dicyano-1,4-*bis*[2-aminophenylthio] butadiene.

## Competing interests

The author(s) declare that they have no competing interests.

## Authors' contributions

ARK, an MSc candidate, participated in the design of the study, performed all the experiments and analysis, prepared the figures, and contributed to the writing of the manuscript. SMB conceived of the study, participated in its design and analysis, and prepared and revised the manuscript. Both authors read and approved the final manuscript.
